# TREM2 Regulates the Removal of Apoptotic Cells and Inflammatory Processes during the Progression of NAFLD

**DOI:** 10.3390/cells12030341

**Published:** 2023-01-17

**Authors:** Imke Liebold, Simon Meyer, Markus Heine, Anastasia Kuhl, Jennifer Witt, Leah Eissing, Alexander W. Fischer, Anja Christina Koop, Johannes Kluwe, Julian Schulze zur Wiesch, Malte Wehmeyer, Uwe Knippschild, Ludger Scheja, Joerg Heeren, Lidia Bosurgi, Anna Worthmann

**Affiliations:** 1 I. Department of Medicine, University Medical Center Hamburg-Eppendorf, 20246 Hamburg, Germany; 2 Protozoa Immunology, Bernhard Nocht Institute for Tropical Medicine, 20359 Hamburg, Germany; 3 Department of Biochemistry and Molecular Cell Biology, University Medical Center Hamburg-Eppendorf, 20246 Hamburg, Germany; 4 Department of Molecular Metabolism, Harvard T. H. Chan School of Public Health, Harvard University, Boston, MA 02115, USA; 5 Department of Cell Biology, Harvard Medical School, Boston, MA 02115, USA; 6 Department of Internal Medicine and Gastroenterology, Amalie Sieveking Hospital, 22359 Hamburg, Germany; 7 Department of General and Visceral Surgery, University Hospital Ulm, 89081 Ulm, Germany

**Keywords:** nonalcoholic fatty liver disease (NAFLD), nonalcoholic steatohepatitis (NASH), metabolic-associated fatty liver disease (MAFLD), macrophages, TREM2, phagocytosis, inflammation, fibrosis, lipoproteins

## Abstract

Nonalcoholic fatty liver disease (NAFLD) is the most common liver pathology worldwide. In mice and humans, NAFLD progression is characterized by the appearance of TREM2-expressing macrophages in the liver. However, their mechanistic contributions to disease progression have not been completely elucidated. Here, we show that TREM2^+^ macrophages prevent the generation of a pro-inflammatory response elicited by LPS-laden lipoproteins *in vitro*. Further, *Trem2* expression regulates bone-marrow-derived macrophages (BMDMs) and Kupffer cell capacity to phagocyte apoptotic cells *in vitro*, which is dependent on CD14 activation. In line with this, loss of *Trem2* resulted in an increased pro-inflammatory response, which ultimately aggravated liver fibrosis in murine models of NAFLD. Similarly, in a human NAFLD cohort, plasma levels of TREM2 were increased and hepatic *TREM2* expression was correlated with higher levels of liver triglycerides and the acquisition of a fibrotic gene signature. Altogether, our results suggest that TREM2^+^ macrophages have a protective function during the progression of NAFLD, as they are involved in the processing of pro-inflammatory lipoproteins and phagocytosis of apoptotic cells and, thereby, are critical contributors for the re-establishment of liver homeostasis.

## 1. Introduction

NAFLDs, or, as recently proposed, “metabolic associated fatty liver diseases” (MAFLD) [1], encompass a broad spectrum of liver pathologies, ranging from simple steatosis to nonalcoholic steatohepatitis (NASH), potentially resulting in liver fibrosis and cirrhosis. NAFLD is common in obese and diabetic people and roughly 25% of the global population is affected by NAFLD [2]. However, up to now, there is no approved drug for the treatment of NAFLD. One important feature of hepatocytes is their ability to efficiently export excess triglycerides and other lipids as triglyceride-rich very-low-density lipoproteins (VLDLs). Thus, an impairment in VLDL secretion, induced by choline-deficient diets or depletion of proteins pivotal for VLDL production, such as apolipoprotein E (APOE), causes liver steatosis [3], the initial step in MAFLD development [4]. Although it is commonly accepted that multiple hits, including lipids as well as gut and adipose tissue-derived signals, may contribute to the development of NAFLD [5], the underlying mechanisms still remain not completely understood. Macrophages are important modulators of the initiation and propagation of hepatic inflammatory and fibrotic processes [6]. So far, it is known that during the progression of NAFLD, the hepatic macrophage compartment undergoes dramatic changes. In particular, in murine models of NAFLD, resident/embryonic T-cell membrane protein 4 (TIM4)^+^ Kupffer cells (KCs) are lost and replenished by monocyte-derived TIM4^−^ macrophages acquiring KC-like features [7,8,9,10]. Interestingly, a subset of these recruited macrophages resembled the expression pattern of triggering receptor expressed on myeloid cells 2 (TREM2)^+^ and cluster of differentiation 9 (CD9)^+^ lipid-associated macrophages (LAMs) [9,10,11], which were recently described in obese adipose tissue [12]. The increased abundance of these TREM2^+^ macrophages in murine NASH as well as in murine and human liver fibrosis was further confirmed in other single-cell transcriptomic datasets [13,14]. Functionally, TREM2^+^ macrophages localized around hepatic crown-like structures [10] and fibrotic areas of the liver [15] and were implicated in lipid handling and fibrotic processes [9,15]. A recent study by Hendrikx and colleagues [15] highlighted that macrophage loss aggravated methionine- and choline-deficient (MCD) diet-induced steatohepatitis. However, detailed mechanistic insights and functional characterization of TREM2^+^ macrophages in systemic metabolic stress, as induced by a hypercaloric diet, are needed to eventually harness these potentially protective cells for therapeutic purposes. Here, using different in vivo murine models of NAFLD and *in vitro* experimental approaches, we investigated whether TREM2 drives hypercaloric feeding-induced NASH progression by modulating macrophage function. We found that TREM2^+^ macrophages are critical for the phagocytosis of dying hepatocytes and attenuate inflammatory responses through the processing of pro-inflammatory lipoproteins. This TREM2^+^ macrophage-mediated effect, in turn, delays fibrosis progression.

## 2. Materials and Methods

### 2.1. Animal Experiments

All animal experiments were approved by the Animal Welfare Officers of University Medical Center Hamburg-Eppendorf (UKE) and Behörde für Gesundheit und Verbraucherschutz Hamburg (G15/66). Mice were bred in-house at UKE (Hamburg, Germany) and housed at 22 °C with a day and night cycle of 12 h. Animals had ad libitum access to food and water. For cell-isolation studies, male C57BL/6J wild-type (WT) *Trem2^+/+^*- mice or *Trem2^−/−^* (*Trem2^tm1(KOMP)Vlcg^*/MbpMmucd) mice were sacrificed at age of 8–12 weeks. To deplete KC, mice were injected intravenously via the tail vein with 200 µL clodronate liposome solution (ClodronateLiposomes.org, Amsterdam, The Netherlands) two days before the experiments, as described before [16]. Triglyceride-rich lipoproteins (TRLs) were isolated from healthy and hyperlipidemic individuals; as described in, *2.15.* 100 µg of TRL (10 mg protein/mL) was injected intravenously via the tail vein.

To assess the uptake of triglyceride-rich lipoprotein (TRL) particles into hepatic macrophages, TRL particles were labeled with DiOC3(3) iodide [3,3-Dipropyloxacarbocyanine iodide] (DID) solution (AAT Bioquest^®^, Inc., Pleasanton, CA, USA, 22,033 at a final concentration of 0.625 mg protein/mL) and 50 µg was injected via the tail vein. Hepatic macrophages were isolated and sorted, as described in *2.4*.

Feeding studies were conducted in age-matched male *Trem2^+/+^*, *Trem2^−/−^*, *Apoe^−/−^* and *Apoe^−/−^Trem2^−/−^* mice starting at age of 8 weeks. *Trem2^+/+^* and *Trem2^−/−^* mice were fed a high-fat diet (HFD) containing sucrose and cholesterol (Ssniff S8301-E020) for 16 weeks. *Apoe^−/−^* and *Apoe^−/−^Trem2^−/−^* mice were fed HFD deficient in choline (Research Diets D05010402) for 26 weeks. Mice were anesthetized after a 4 h fasting period and blood for EDTA plasma was withdrawn transcardially. Subsequently, animals were perfused with PBS, tissues were harvested and snap frozen with liquid nitrogen and immediately conserved either in TRIzol^®^ reagent (Invitrogen, MA, USA) or stored at −80 °C for further processing.

### 2.2. Human Data

For correlative analysis, log-transformed hepatic gene expression of *TREM2 (*log* TREM2)* and *COL1A1 (*log* COL1A1)* data as well as metabolic parameters from patients of different body-mass index (BMI) were included in a biobank study conducted at the Department of General and Visceral Surgery, University of Ulm, Germany from 2002 to 2010, previously described in detail [17]. Briefly, people (*n* = 120) undergoing bariatric surgery (*n* = 57), abdominal surgery for early colorectal cancer (*n* = 34), pancreatic cancer (*n* = 18), other cancers of the gastrointestinal tract or benign diseases, such as sigma-diverticulosis, were included in the study. Exclusion criteria were type 1 diabetes and acute inflammatory disease. None of the cancer patients exhibited signs of metastases or reported excessive, unintended recent weight loss. Liver biopsies and blood samples were immediately placed on ice and subsequently snap frozen and stored in liquid nitrogen or at −80 °C.

Plasma TREM2 levels were measured after overnight fasting in patients with biopsy-proven NAFLD and liver-healthy control subjects, who were characterized by normal liver function tests, low liver elastography and controlled attenuation parameter measurements (FibroScan; Echosens, Paris, France). The study was approved by the local ethics committee in Hamburg, Germany (PV5036), and complied with the 1975 Declaration of Helsinki. Written, informed consent was obtained from each subject.

### 2.3. Gene Expression

Total RNA was isolated and purified from organs and cells using NucleoSpin RNA II kit (Macherey & Nagel, Düren, Germany) and cDNA was prepared using SuperScript^®^ III Reverse Transcriptase (Invitrogen). Human liver RNA samples and cohorts were published previously [17]. Quantitative real-time PCR reactions for indicated genes were conducted on a 7900HT sequence detection system (Applied Biosystems, Waltham, MA, USA) using TaqManAssay-on-Demand primer sets (Applied Biosystems, *COL1A1*: Hs00164004_m1, *TAF*: Hs00270322_m1, *TREM2*: Hs01010721_m1, *Ccl2*: Mm00441242_m1, *Cd36*: Mm00432403_m1, *Col1a1*: Mm00801666_g1, *Il1b*: Mm00434228_m1, *Il6*: Mm00446190_m1, *Lipa*: Mm01204975_m1, *Lpl*: Mm00434764_m1 *Tbp*: Mm00446973_m1, *Tgfb1*: Mm00441724_m1, *Tnf*: Mm00443258_m1, *Trem2*: Mm00451744_m1,). Cycle thresholds (Cts) were normalized to the housekeeper TATA-box-binding protein (Tbp) or TATA-box-binding protein-associated factor 1 (TAF1).

### 2.4. Cell Isolation/Cell Culture

To isolate KC, 8–12-week-old mice were perfused with collagenase solution (Collagenase NB4G, Serva, 0.05% in GBSS) via the portal vein. The liver was removed, shred and incubated at 30 °C to allow for digestion. After filtering the solution (100 µm), parenchymal cells were removed by centrifugation (2 × 4 min at 40× *g*). Non-parenchymal cells were pelletized and erythrocytes were eliminated by Ammonium-Chloride-Potassium Lysing Buffer. Cells were enriched in the interphase by Optiprep™-PBS centrifugation (25 min at 400× *g*) and incubated with MACS buffer (0.5 mM DTA, 0.5% BSA in PBS), mouse IgG (15 min, 4 °C) and Fc-block (10 min 4 °C; BD Biosciences, Heidelberg, Germany). KCs (anti-F4/80) were isolated using MACS technology according to manufacturer instructions (Milteny Biotech, Bergisch Gladbach, Germany).

### 2.5. Bone-Marrow-Derived Macrophage Differentiation

For the differentiation of bone-marrow-derived macrophages (BMDMs), hematopoietic pluripotent stem cells were isolated from bone marrows of *Trem^+/+^* or *Trem^−/−^* mice and differentiated in cRPMI (10% FCS, 15% L929 supernatant, 200 mM L-Glu and 0.5% Gentamycin). On day 3, half of the medium was replaced, while on day 5, cells were split and seeded into fresh media (10 mL). Fully differentiated macrophages were harvested on day 7, seeded into a 24-well plate (0.33 × 10^6^ cells/well) and used for experiments.

BMDMs were then stimulated with LPS (100 ng/mL) for 4 h (to measure gene expression) or for 18 h (to determine cytokine secretion).

### 2.6. Phagocytosis Assays

Phagocytosis assay was performed using primary liver macrophages. Therefore, liver cells were isolated, as described in 2.4. Without lysating the red blood cells, the non-parenchymal fraction was diluted in 7–10 mL of pre-warmed DMEM (5% FCS, 200 mM L-Glu and 0.5% Gentamycin). Afterward, cells were plated in Cell+ 12-well plates (Sarstedt, Nümbrecht, Germany) overnight in 1.5 mL media per well. On the next day, cells were gently washed to remove non-adherent cells, examined for their morphological phenotype under a microscope and supplemented with fresh warm DMEM. After 24 h, liver macrophages were used for further analysis. First, apoptosis was induced in hepatocytes (Hepa 1–6 cell line) via heating. Briefly, hepatocytes were washed and harvested in PBS with a cell scraper. Subsequently, cells were incubated at 40 °C for 45 min while shaking. Thereafter, apoptosis was verified via Annexin V and Prodium Iodide (PI) staining, using the Apoptotic Detection Kit (BioLegend, London, UK)

The apoptotic hepatocytes (aHs) were counted and stained with CellTrace™CSFE (Thermo Fisher Scientific, Massachusetts, USA) in a final concentration of 10 × 10^6^ cells/mL, before performing the phagocytosis assay at 37 °C and at 4 °C or in the presence of Cytochalasin D (Cyt.D). The plated BMDMs or KCs were incubated with 0.66 × 10^6^ aHs in a ratio of 1:2 for 45 min. Afterward, cells were washed 5× with PBS to remove unbound/not-phagocytosed aHs and stained with F4/80 (AF700) and CD11b (APC-Cy7). The phagocytosis rate was examined at the LSR II (BD Biosciences). The ratio between macrophages that are positive for the aH-dye (CSFE) at 37 °C and 4 °C/Cyt.D defines the phagocytic rate of the macrophages.

### 2.7. CD14 Assay

In some experiments, the phagocytosis assay was performed in the presence of anti-CD14 or the IgG control. KCs (isolated as described in 2.4) were incubated with 10 µg of the anti-CD14 antibody (BD Biosciences, Heidelberg, Germany) or the IgG control (BD Biosciences, Heidelberg, Germany) for 2 h. Subsequently, cells were stimulated with 10 ng/mL of LPS for 20 h. The phagocytosis assay was performed afterward, as described previously.

### 2.8. Liver Immunophenotyping

For the immunophenotyping, the liver was perfused manually with 10 mL of PBS. The liver was intersected and the gallbladder removed before mincing the liver. The liver suspension was incubated for 45 min at 37 °C while shaking in digestion buffer (DMEM, 1 mg/mL Collagenase IV (StemCell, Cologne, Germany), 150 U/mL DNaseI, 0.2 M MgCl_2_, 0.5 M CaCl_2_). Subsequently, the cell suspension was filtered through a 70 µm filter and washed 3 times with PBS (300× *g* 5 min, 4 °C). To remove the hepatocytes and to obtain the non-parenchymal fraction, the cell suspension was centrifuged at 50xg for 4 min. The pellet was discarded and the supernatant harvested. The cells were pelleted and red blood cells lysated (3 mL, 5 min, RT). Cells were then washed, incubated with Fc block (BioLegend, London UK, anti-CD16/CD32 1:100,000) for 15 min at 4 °C prior to the staining of surface epitopes. For the staining of surface epitopes, the cells were incubated with the antibody cocktail for 35 min at 4 °C in the dark. For the analysis of intracellular epitopes, cells were fixed and permeabilized using the Foxp3/Transcription Factor Staining Buffer Set (eBioscience, Massachusetts, USA) and stained afterward for 35 min at 4 °C in the dark. Cells were analyzed at the LSRII (BD Biosciences, Heidelberg, Germany). The data were analyzed using the FlowJo software (BD).

### 2.9. Apoptosis Detection

For the detection of Annexin V^+^ apoptotic cells in the liver, cells were isolated as described above and stained with an antibody cocktail for labelling surface epitopes. Afterward, cells were washed twice with PBS + 2% FCS and then with Annexin V binding buffer. Thus, 200 µL of Annexin V binding buffer was added to the cells, followed by 2 µL of FITC-Annexin V per sample. The cells were incubated for 15 min in the dark at room temperature. Subsequently, samples diluted in 200 µL of Annexin V binding buffer were analyzed at the flow cytometer.

### 2.10. Western Blotting

After organ harvesting, pieces of liver were snap frozen in liquid nitrogen. To generate protein lysates for SDS-PAGE, liver samples were homogenized using RIPA buffer supplemented with protease inhibitors (Roche). Protein samples (50 µg per lane) were separated on a 10% Bis-Tris (pH 6.6) polyacrylamide gel using NuPAGE^®^ MES SDS Running Buffer under reducing conditions (Invitrogen, Massachusetts, USA). After the transfer to nitrocellulose membranes, blots were blocked for 2 h with 5% w/v nonfat dry milk in 1X TBST and incubated with the primary antibody against phosphorylated c-Jun N-terminal kinase (JNK)-Protein (Cell Signaling 1:250, Cat. No 9251), total JNK (1:1000, Cat. No. 9252), CASPASE 3 (Cell Signaling Technology (8G10)) and cleaved CASPASE 3 (Cell Signaling Technology (5AE1)), TUBULIN (Abcam, ab179503) and against mouse GAPDH (Novus, 1:1500, Cat. No. NB300-320) overnight at 4 °C. Membranes were incubated for 1.5 h in secondary horseradish peroxidase-conjugated antibody solutions (goat anti-rabbit, Jackson Immunoresearch, 1:5000, Cat. No. 111-035-144). Protein bands were detected using a luminol and para-hydroxycoumarinic acid-based chemiluminescence substrate. Densitometric quantification was performed using the control software Amersham Imager 600.

### 2.11. Plasma Analysis of Alanine Aminotransferase and LPS

Liver transaminase plasma activity was assessed by automated measurement of alanine aminotransferase (ALT) using a COBAS Mira System (Roche, Mannheim, Germany). LPS in serum and isolated TRLs was assessed using the LAL-Kit (Hycultech, Beutelsbach, Germany).

### 2.12. ELISA

Cytokine secretion in cell supernatants was determined using commercial kits (IL 6: STD, Capture- and Detection- Antibody; R&D Cat: No. 840115,840114, 840113), (IL1β: Capture- and Detection- Antibody R&D Systems Cat: No. 840115,840114, 840113) and TNFα (Capture- and Detection- Antibody R&D Systems Cat: No. 840115,840114, 840113 DuoSt, R&D) according to manufacturer’s instructions.

TREM2 concentrations in human plasma samples were determined using DuoSet^®^ ELISA for Human TREM2 from R&D Systems (DY1828-05) according to manufacturer instructions.

### 2.13. Liver Histology

Organ samples for histology were collected in 3.7% formaldehyde solution in PBS. Organs were embedded in paraffin and slides were stained with hematoxylin and eosin (H&E), Sirius Red staining or F4/80 Ab (AbD Serotec, MCA497) staining via immunohistochemistry, according to standard protocols.

### 2.14. Liver Triglyceride Measurements

Liver tissue was homogenized in PBS (10 µL/mg). Liver triglycerides were determined directly from the suspension by using a commercial kit from Roche according to the manufacturer’s instructions.

### 2.15. Isolation of Triglyceride-Rich Lipoprotein (TRLs) from Plasma

TRLs were isolated from plasma via density gradient ultracentrifugation. Blood plasma from patients was mixed with a 60% sucrose solution to yield an end concentration of 10% sucrose. Polyallomer tubes (for SW41 rotors, Beckman) were filled with NaCl (ad 12 mL (total volume of tubes)) and the plasma–sucrose mix was carefully added underneath. Centrifugation was carried out at 4 °C and 28.000 rpm for 3 h. TRLs were then taken from the upper phase.

### 2.16. Statistics

Data are expressed as mean ± S.E.M. As stated in the figure legends, depending on the experimental setup, statistically significant differences between experimental groups were assessed using Mann–Whitney test, student’s *t* test and two-way ANOVA. Association of hepatic *TREM2* expression with liver parameters was performed using Pearson’s correlation coefficient. All statistical analyses were performed using GraphPad Prism 9 and statistically significant differences are depicted by *p*-values or as different letters.

## 3. Results

### 3.1. Lipoproteins Evoke a Macrophage-Dependent Pro-Inflammatory Response

**Figure 1:**
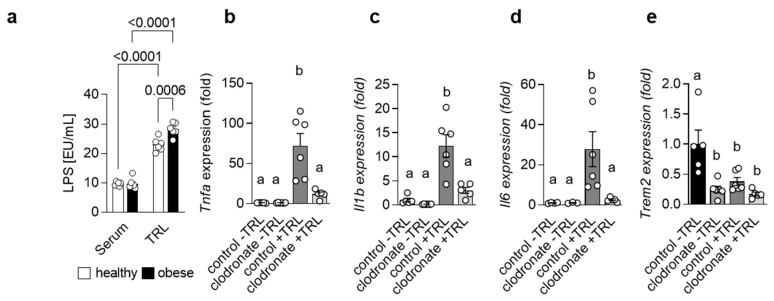
LPS-containing TRLs elicit a macrophage-dependent pro-inflammatory response in the liver (**a**) LPS levels were measured in serum and TRL particles isolated from healthy and obese patients (*n* = 6). (**b**–**e**) Mice were pre-treated or not with clodronate to deplete macrophages and then injected without or with LPS-containing TRL particles. Liver tissue was harvested 4 h after injection. Hepatic gene expression of *Tnfa* (**b**), *Il1b* (**c**), *Il6* (**d**) and *Trem2* (**e**) (*n* = 5–6). Data are shown as mean + SEM. Statistically significant differences between groups (*p* < 0.05) were determined by two-way ANOVA and are indicated as *p*-value (**a**) or as different letters (**b**–**e**).

Adipose tissue as well as gut-derived signals are implicated in the progression of NAFLD [5]. In fact, it has been shown that the severity of NAFLD is associated with alterations in the intestinal microflora and even dysbiosis [18]. In line, higher endotoxin levels have been reported in patients suffering from NASH [19,20]. Lipopolysaccharide (LPS), the major endotoxin, induces a pro-inflammatory response via its receptor Toll-like receptor 4 (TLR4) [21]. Interestingly, mice with impaired TLR4 signaling show reduced NASH after high-fat diet (HFD) feeding and TLR4 protein levels are altered in patients with NASH, suggesting an important role for LPS in the progression of NASH. Previously, it was reported that high-fat meals increase plasma LPS levels [22,23,24] and that in mice, LPS may be transported in the circulation via Triglyceride-rich lipoproteins (TRLs) [25]. Indeed, when we measured LPS levels in serum and in isolated TRL particles from human healthy controls and obese patients, we detected substantial higher amounts of LPS in the TRL particles compared to the serum. Of note, TRL from obese patients had a higher LPS load compared to controls (Figure 1a), indicating that, during obesity, LPS-containing TRL particles may contribute to systemic and, most likely, hepatic inflammation. The uptake of lipoproteins into macrophages has been studied extensively in the context of atherosclerosis, where macrophages in the intima take up oxidized low-density lipoprotein (LDL) particles, become foam cells and promote atherogenesis [26]. However, recently, it was shown that TRL can also be internalized by BMDMs [27] and, more importantly, also by KC in the liver [28]. We hypothesized that during the progression of NASH, LPS-enriched TRLs are taken up by hepatic macrophages, thereby driving a pro-inflammatory response in the liver. To address this hypothesis, we injected TRL particles isolated from lean and obese/hyperlipidemic individuals into wild-type mice. TRL particles elicited an acute pro-inflammatory response in the liver, as indicated by higher hepatic expression of *Tnfa*, *Il1b* and *Il6* (Figure 1b–d). Most importantly, the generation of a pro-inflammatory environment was mainly driven by hepatic macrophages, as in mice pre-treated with clodronate liposomes, the depletion of phagocytic cells led to a reduced pro-inflammatory response (Figure 1b–d). Of note, following both TRL injection as well as clodronate treatment, hepatic gene expression of *Trem2* was reduced (Figure 1e). Overall, these data suggest that in the context of obesity and NAFLD, hepatic macrophages induce a pro-inflammatory response via sensing LPS-containing TRL particles.

### 3.2. Trem2 Controls TRL-Induced Pro-Inflammatory Response

**Figure 2:**
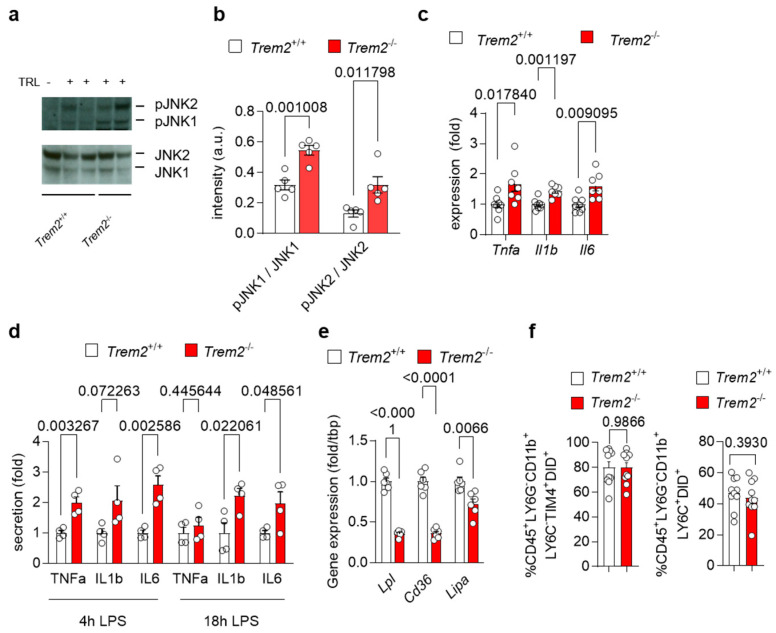
*Trem2* controls LPS- and TRL-induced hepatic pro-inflammatory response (**a**–**c**) WT (*Trem2^+/+^*) and *Trem2^−/−^* mice were injected with TRL particles. (**a**) Western blot of JNK1 and JNK2 as well as phosphorylated JNK1 and JNK2 and (**b**) its quantification (*n* = 4). (**c**) Hepatic gene expression of *Tnfa*, *Il1b* and *Il6* (*n* = 7). (**d**) KC from WT (*Trem2^+/+^*) and *Trem2^−/−^*mice were treated with LPS and secretion of TNFα, IL1β and IL6 into the media was measured after 4 and 18 h (*n* = 4). (**e**) Gene expression of *Lpl*, *Cd36* and *Lipa* in WT (*Trem2^+/+^*) and *Trem2^−/−^* BMDMs. (**f**) Uptake of DiD-labeled TRL particles into WT (*Trem2^+/+^*) and *Trem2^−/−^* macrophages (*n* = 9–10). Data are shown as mean + SEM. Statistically significant differences between groups (*p* < 0.05) were determined by Student’s *t*-test (**a**–**e**) or Mann–Whitney test (**f**) and are indicated as *p*-value.

To investigate how *Trem2* regulates the generation of a pro-inflammatory environment upon liver exposure to lipoproteins, we first injected WT (*Trem2^+/+^)* and *Trem2^−/−^* mice with TRL particles and evaluated the activation status of the liver. Upon TRL injection, hepatic protein levels of the phosphorylated and active form of the stress kinases JNK1 and JNK2, markers of liver fibrosis [29], were elevated in *Trem2*^−/−^ mice compared to WT mice (Figure 2a–b). In the same line, expression of pro-inflammatory cytokines, such as *Tnfa*, *Il1b* and *Il6,* was higher in *Trem2*^−/−^ mice compared to WT mice (Figure 2c). We also confirmed these findings in vitro, where treatment of *Trem2^−/−^* KCs with LPS resulted in higher secretion of the pro-inflammatory cytokines TNFα, IL1β and IL6 compared to the control counterpart (Figure 2d). Overall, these data demonstrate that loss of *Trem2* results in an exacerbated pro-inflammatory response to LPS-loaded TRLs, hence, indicating a liver protective role for *Trem2* during the progression of NASH.

Next, we investigated the mechanisms of how *Trem2* expression might regulate the inflammatory response in the liver. As TREM2*^+^* macrophages are well known as LAM [12], which arise in the liver upon HFD feeding [9,10] and have been involved in lipid handling [15], we first analyzed the expression of genes involved in lipid and lipoprotein uptake and processing [30] in WT and *Trem2^−/−^* BMDMs. Interestingly, *Trem2^−/−^* BMDMs showed reduced mRNA levels of lipoprotein lipase (*Lpl)* and the cluster of differentiation 36 *(Cd36)*, a scavenging receptor involved in fatty acid uptake (Figure 2e). Similarly, expression of *Lipa* encoding lysosomal acid lipase was decreased in *Trem2^−/−^* BMDMs compared to the control counterpart (Figure 2e), suggesting the contribution of *Trem2* for lipoprotein and fatty acid uptake subsequent processing. Next, we studied whether the loss of *Trem2* affected KC-dependent uptake of TRL particles. For this purpose, we injected WT (*Trem2^+/+^*) and *Trem2^−/−^* mice with DiD-labeled TRL particles and quantified their uptake into KCs (gated as CD45^+^LY6G^−^CD11b^+^LY6C^−^TIM4^+^) and infiltrating macrophages (gated as CD45^+^LY6G^−^CD11b^+^LY6C^+^) via flow cytometry. Interestingly, in this setting, *Trem2^−/−^* macrophages showed a similar capacity to uptake TRL particles as the control counterpart (Figure 2f).

Together, these data suggest that in response to TRLs, loss of *Trem2* leads to the generation of a pro-inflammatory environment, without affecting the clearance of these particles by hepatic macrophages.

### 3.3. Trem2 Regulates KC-Dependent Uptake of Apoptotic Cells

**Figure 3:**
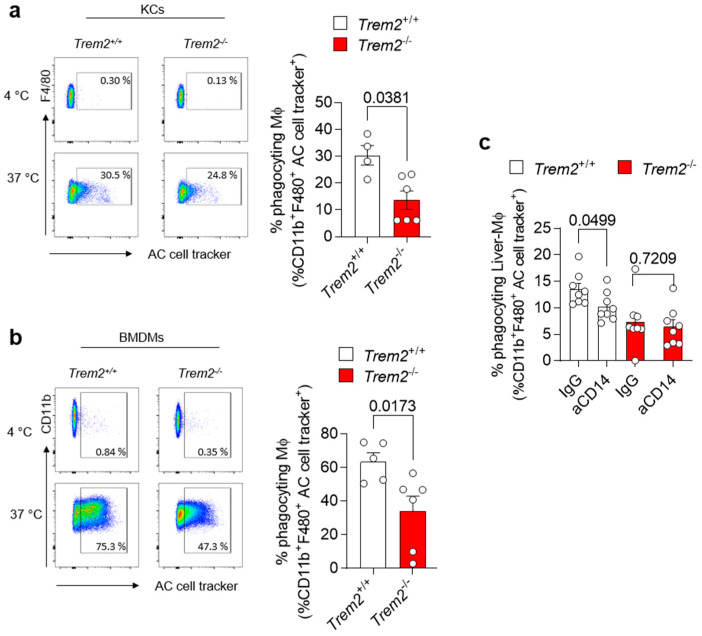
*Trem2* regulates KC-dependent uptake of apoptotic cells (**a**) KCs were isolated from WT (*Trem2^+/+^*) and *Trem2^−/−^* mice and incubated with labeled aHs at 4 °C (binding control) and 37 °C (binding+uptake); (left panel): gating strategy, (right panel): quantification of phagocyting macrophages (*n* = 4–5). (**b**) BMDMs were isolated from WT (*Trem2^+/+^)* and *Trem2^−/−^* mice and incubated with labeled apoptotic hepatocytes at 4 °C (binding control) and at 37 °C (uptake); (left panel): gating strategy (right panel): quantification of phagocyting macrophages (*n* = 5–6). (**c**) Hepatic macrophages were isolated from WT (*Trem2*^+/+^) and *Trem2^−/−^* mice, pre-treated with control (IgG) or anti-CD14 antibody (aCD14), stimulated with LPS for 20 h and incubated with labeled aHs. Phagocyting macrophages were quantified (*n* = 8). Data are shown as mean + SEM. Statistically significant differences between groups (*p* < 0.05) were determined by Mann–Whitney test (**a**,**b**) and Student’s *t*-test (**c**) and are indicated as *p*-value.

Accumulation of apoptotic cells due to impairment in their clearance by efferocytic macrophages is one of the processes leading to the generation of a pro-inflammatory environment. *Trem2* expression by microglial cells has been described to be implicated in the phagocytosis of apoptotic neurons [31]. However, whether TREM2 engagement regulates efferocytosis in the damaged liver has not been completely dissected so far. Thus, we isolated KCs from the livers of WT (*Trem2^+/+^*) and *Trem2^−/−^* mice and exposed them to aHs. Similarly, BMDMs, which resemble the cells infiltrating the liver upon damage, were incubated with dying cells, and their phagocytic capacity was assessed. In both scenarios, *Trem2* regulated the capacity of resident and infiltrating phagocytes to clear aHs (Figure 3a,b). These data highlight the critical role of TREM2 as a phagocytic receptor in bone-marrow-derived and hepatic macrophages.

The synergistic interaction of receptors involved in phagocytosis has been described as a strategy to ensure efficient and prompt clearance of pathogens and potentially harmful dying cells in various experimental settings [32,33]. Given the impact of *Trem2* on LPS-mediated response (Figure 2d) and the role of CD14, an LPS-binding protein, in regulating phagocytosis directly or in cooperation with other receptors [34,35], we tested whether synergy between TREM2 and the LPS co-receptor CD14 [36] occurs and whether it contributes to the regulation of phagocytosis. We thus treated WT (*Trem2^+/+^*) and *Trem2^−/−^* hepatic macrophages with anti-CD14 or isotype control (IgG) antibodies, prior to their stimulation with LPS. Afterwards, these LPS-activated macrophages were exposed to apoptotic cells and their phagocytic capacity was evaluated. Similar to what is described in human monocyte-derived macrophages [34], treatment of WT KCs with anti-CD14 diminished their capacity to phagocyte apoptotic cells. In contrast, no further reduction in the uptake of apoptotic cells was observed in *Trem2^−/−^* KCs pre-treated with anti-CD14 antibody compared to IgG (Figure 3c and Figure S1). These data suggest that TREM2 and CD14 act in concert to mediate LPS-induced phagocytosis.

### 3.4. Loss of Trem2 Aggravates Liver Damage in a Murine Model of NASH

**Figure 4:**
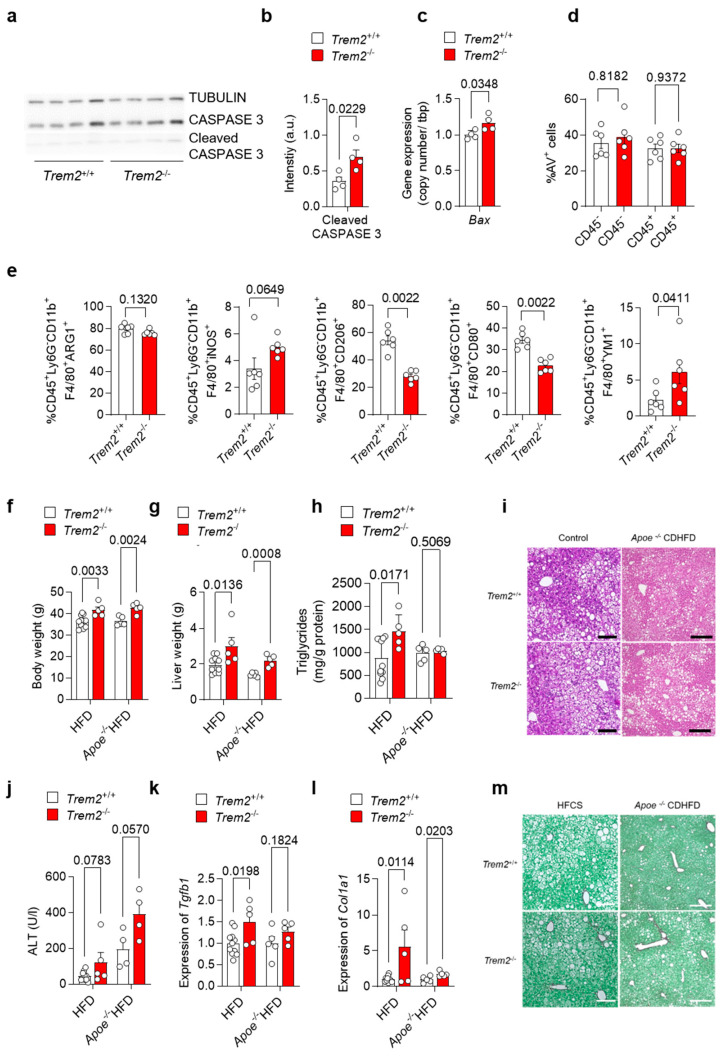
Loss of *Trem2* results in the accumulation of apoptotic cells and aggravates liver damage. (**a**) Western Blotdepicting CASPASE 3 and cleaved CASPASE 3 levels as a marker for hepatic apoptosis and (**b**) quantification of cleaved CASPASE 3 expression (*n* = 4) and (**c**) gene expression of *Bax* performed on liver samples isolated from of WT (*Trem2^+/+^*) and *Trem2^−/−^* mice (*n* = 4). WT (*Trem2^+/+^*) and *Trem2^−/−^* mice were fed with an HFD and (**d**) the frequency of hepatic apoptotic cells detected by Annexin V staining (*n* = 6), and (**e**) frequency of differentially polarized macrophage populations is shown (*n* = 6). (**f**–**m**). WT (*Trem2^+/+^*) and *Trem2^−/−^* mice as well as WT (*Trem2^+/+^*) and *Trem2^−/−^* mice deficient for *Apoe* were fed a HFD for 16 weeks. (**f**) Body weight, (**g**) liver weight, (**h**) liver triglyceride (TG) levels, (**i**) H&E staining of liver sections (scale bar = 200 µm), (**j**) plasma ALT levels, hepatic gene expression of (**k**) *Tgfb*1 and (**l**) *Col1a1* (*n* = 5–8) and (**m**) Sirius red staining of liver sections (scale bar = 200 µm). Data are shown as mean + SEM. Statistically significant differences between groups (*p* < 0.05) were determined by Student’s *t*-test (**b**,**c**,**f**–**h**,**j**–**l**) and Mann–Whitney test (**d**,**e**) and are indicated as *p*-value.

As our data suggest an important role for *Trem2* in the KC-mediated clearance of dying cells as well as for the development of a pro-inflammatory response, we next aimed to study the consequences of *Trem2* deficiency during the progression of NAFLD. In a first step, we evaluated the levels of apoptotic-cell-associated proteins in livers derived from WT (*Trem2^+/+^*) and *Trem2^−/−^* mice in steady-state conditions. In line with the reduced phagocytic capacity of *Trem2^−/−^* KC, a higher amount of cleaved CASPASE 3 protein and a higher gene expression of pro-apoptotic *Bax* were detected in mice lacking *Trem2* compared to controls (Figure 4a–c). Next, to assess the hepatic accumulation of apoptotic cells during the progression of NAFLD, we fed WT (*Trem2^+/+^*) and *Trem2^−/−^* mice with an HFD for 2 weeks. In this experimental setting, the amount of apoptotic cells, as measured by Annexin V staining (AV^+^ cells), remained similar in WT vs. *Trem2^−/−^* mice (Figure 4d), thus suggesting that compensatory mechanisms leading to the uptake of dying cells occur in the absence of *Trem2* during a short-term HFD. Additionally, although no statistically significant differences were observed in the frequency of macrophages expressing ARG1 or iNOS, prototypical anti- and pro-inflammatory markers respectively, isolated from the liver of HFD-fed WT (*Trem2^+/+^*) and *Trem2*^−/−^ mice, a reduction in the percentage of a “metabolically active” CD206^+^ macrophage population [37] was detected in *Trem2*^−/−^ mice. In parallel, reduced expression of the costimulatory molecule CD80 and increased expression of the anti-inflammatory but also pro-fibrotic protein YM1 was detected in *Trem2*^−/−^ macrophages compared to controls (Figure 4e).

Additionally, we analyzed the role of TREM2 in long-term models of NALFD induced either by an HFD, which causes accumulation of F4/80^+^ macrophages in the damaged liver (Figure S2), or by the combination of *Apoe* deletion with HFD. Here, we observed that mice lacking *Trem2* had higher body and liver weight (Figure 4f–g), which is in line with increased body-weight gain described previously [12]. Further, when fed a HFD, *Trem2^−/−^* mice had increased liver triglyceride levels (Figure 4h), suggesting hepatic steatosis as also visible in H&E-stained liver sections (Figure 4i). In line with this, plasma ALT levels, as a measure of liver damage, were increased by trend in *Trem2^−/−^* mice (Figure 4j). Similarly, hepatic expression of pro-fibrotic genes *Tgfb1* and *Col1a1* was increased in *Trem2^−/−^* mice compared to WT (*Trem2^+/+^*) (Figure 4k-l) and higher hepatic fibrosis was also revealed by Sirius Red staining (Figure 4m), which is in line with previous studies [15]. In sum, these data suggest that loss of *Trem2* results in an altered immune response, most likely contributing to higher liver fibrosis during the progression of NAFLD.

### 3.5. Trem2 in Human NAFLD

**Figure 5:**
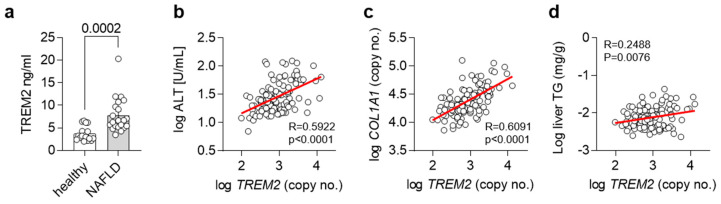
TREM2 in human NAFLD. (**a**) Levels of soluble TREM2 in human healthy controls or NASH patients (*n* = 19–21). (**b**–**d**) Correlation of hepatic *TREM2* expression with (**b**) plasma ALT levels, (**c**) hepatic *COL1A1* and (**d**) liver triglyceride (TG) levels in a human cohort (*n*= 120). (**a**) Data are shown as mean + SEM. Statistically significant differences between groups (*p* < 0.05) were determined by Student’s *t*-test and are indicated as *p*-value. (**b**–**d**) Correlation is given as Pearson R.

As we and others have shown an important regulatory role for *Trem2* and TREM2^+^ macrophages in the progression of NAFLD in mice [15], we next wanted to translate our findings into humans. For this purpose, we compared levels of TREM2 in plasma from healthy controls as well as NALFD patients. In accordance with our findings, plasma levels of TREM2 were elevated in NALFD patients (Figure 5a). In a separate cohort, we correlated hepatic gene expression levels of *TREM2* with plasma ALT levels, as a measure of liver damage (Figure 5b), hepatic gene expression levels of *COL1A1*, as a liver fibrosis marker (Figure 5c), and with liver triglyceride (TG) levels, as a marker for hepatic steatosis (Figure 5d). Hepatic gene expression of *TREM2* correlated with all the investigated features of NAFLD, overall suggesting that also in humans, TREM2 might be implicated in the development of NAFLD and its progression into NASH.

## 4. Discussion

While the origin and transcriptional profile of macrophage populations emerging in the liver upon NAFLD have been investigated thoroughly [7,8,9,10] and resulted in the identification of a TREM2^+^ subset, which is either termed LAM- [9,12] or NASH-associated macrophages (NAMs) [13] and Scar-associated macrophages (SAMs) [14], respectively, little is known about the mechanisms promoting the protective role of these TREM2^+^ macrophages. Only very recently, Hendrikx and colleagues were able to show that TREM2^+^ macrophages are implicated in hepatic fibrosis and that loss of *Trem2* aggravated murine MCD-induced NASH [15]. The here-presented data provide evidence that, mechanistically, during the progression of NASH, *Trem2* expression, in addition to regulating the pro-inflammatory responses to TRL particles by liver macrophages, also affects their capacity to clear apoptotic cells in the damaged liver.

As NASH progression is believed to be linked to gut dysbiosis and gut-derived signals, and especially LPS and its receptor TLR4 [38,39,40], we first investigated if gut-derived TRL particles might be containing LPS in obesity and NAFLD. Indeed, in line with other studies highlighting the role of high-fat-meal-induced endotoxemia [22,23,24], we found increased levels of LPS in TRL particles isolated from obese patients. Of note, upon injection of these particles into mice, they were able to induce a KC-dependent pro-inflammatory response in the liver. Thus, the lipoprotein-mediated delivery of bacterial LPS might be a novel mechanism of how gut-derived signals promote liver inflammatory processes. Indeed, in other tissues, the uptake of low-density lipoproteins by macrophages has been shown to modulate inflammatory responses and contribute to overall disease progression [26] and, only very recently, it has also been shown that the uptake of TRL particles by BMDMs might influence pro-inflammatory gene expression [27].

Next, we were able to show that taming the inflammatory response upon injection of LPS-containing TRLs was attenuated by TREM2, as mice lacking *Trem2* showed higher expression and secretion of pro-inflammatory cytokines.

Preventing the generation of a pro-inflammatory environment, on the one hand, may be explained by a direct effect of LPS on TREM2 [41], which, in turn, signals through the adaptor protein DAP12 (TYROBP) to restrain pro-inflammatory cytokine production [42]. In parallel to this, in our experimental settings, TREM2 expressed by hepatic macrophages may bind to TRLs and inhibit the secretion of pro-inflammatory cytokines, such as IL-6. This binding may be similar to what has been described in the context of Alzheimer’s disease, where TREM2 in microglia binds to amyloid-β–lipoprotein complexes via APOE, thereby preventing amyloid-β accumulation [43].

On the other hand, an anti-inflammatory effect of TREM2 can be linked to TREM*2*^+^-BMDMs and KC capacity to clear dying hepatocytes in the liver. The phagocytic process occurring upon engagement of TREM2, similar to what is described for the uptake of apoptotic neurons [31,44] and bacterial products [45], leads to the elimination of the pathogen and of potentially harmful dying cells. This clearance is associated with a reduced pro-inflammatory response [46] and with the induction of a tissue remodeling program essential for the re-establishment of homeostasis [47]. In line with these findings, we and others provide indications for higher apoptosis or decreased macrophage phagocytic capacity in the livers of mice lacking *Trem2* [15,48], factors which drive the progression of NASH.

Moreover, the deletion of *Trem2* resulted in increased weight gain, liver damage and hepatic fibrosis in two different models of NAFLD. This is in line with a previous work reporting that loss of *Trem2* causes an exacerbated steatohepatitis, cell death and fibrosis in an MCD-induced NASH model [15]. Similarly, in the context of other liver pathologies, such as acute liver injury, primary sclerosing cholangitis and hepatocellular carcinoma, TREM2 has been shown to exert a protective function [49,50,51]. Hence, there is an urgent need for future studies to clarify if and how TREM2^+^ macrophages might be used therapeutically to prevent the progression of NAFLD and other liver pathologies. In this scenario, a deeper understanding of how the cooperation between TREM2 and CD14 occurs, as also confirmed in a recent preprint [52], might help to design better future therapies with TREM2-expressing macrophages. In particular, whether a physical interaction between the two receptors occurs and its meaning in physiological conditions still needs to be evaluated.

Finally, in addition to generating mechanistic insights into the protective function of TREM2^+^ macrophages in mice, we also provide human data on TREM2 in the context of NALFD. We confirm previously published data [15] on increased levels of soluble TREM2 in the plasma of human NASH patients and show a strong positive correlation of hepatic *TREM2* expression with markers of liver damage. Whether higher TREM2 might arise due to the cleavage of TREM2 by ADAM17 [53] or is the result of an increased amount of circulating TREM2^+^ macrophages, remains to be clarified. Additionally, whether this potentially soluble TREM2 exerts a protective function during NASH has to be addressed in further studies. Nevertheless, based on our and previous findings [15], plasma levels of TREM2 seem to be a valid marker for human NAFLD, likely correlated with disease severity. Plasma TREM2 should be validated in the future as an easy non-invasive biomarker to assess NAFLD severity.

## Data Availability

The data presented in this study will be made openly available via MendeleyXXXX.

## Supplementary Materials

The following supporting information can be downloaded at: https://www.mdpi.com/article/10.3390/cells12030341/s1, Figure S1: Synergy between TREM2 and CD14 regulates phagocytosis of apoptotic hepatocytes by liver macrophages. Figure S2: Hepatic macrophage infiltration after HFD feeding.
