# Supplementary material for: TREM2 Regulates the Removal of Apoptotic Cells and Inflammatory Processes during the Progression of NAFLD
Source: Cells. 2023 Jan 17;12(3):341. doi: 10.3390/cells12030341 (PMC9913311; doi:10.3390/cells12030341)
Supplement: Supplementary file 1 [file cells-12-00341-s001.zip › cells-2133151-supplementary.pdf]

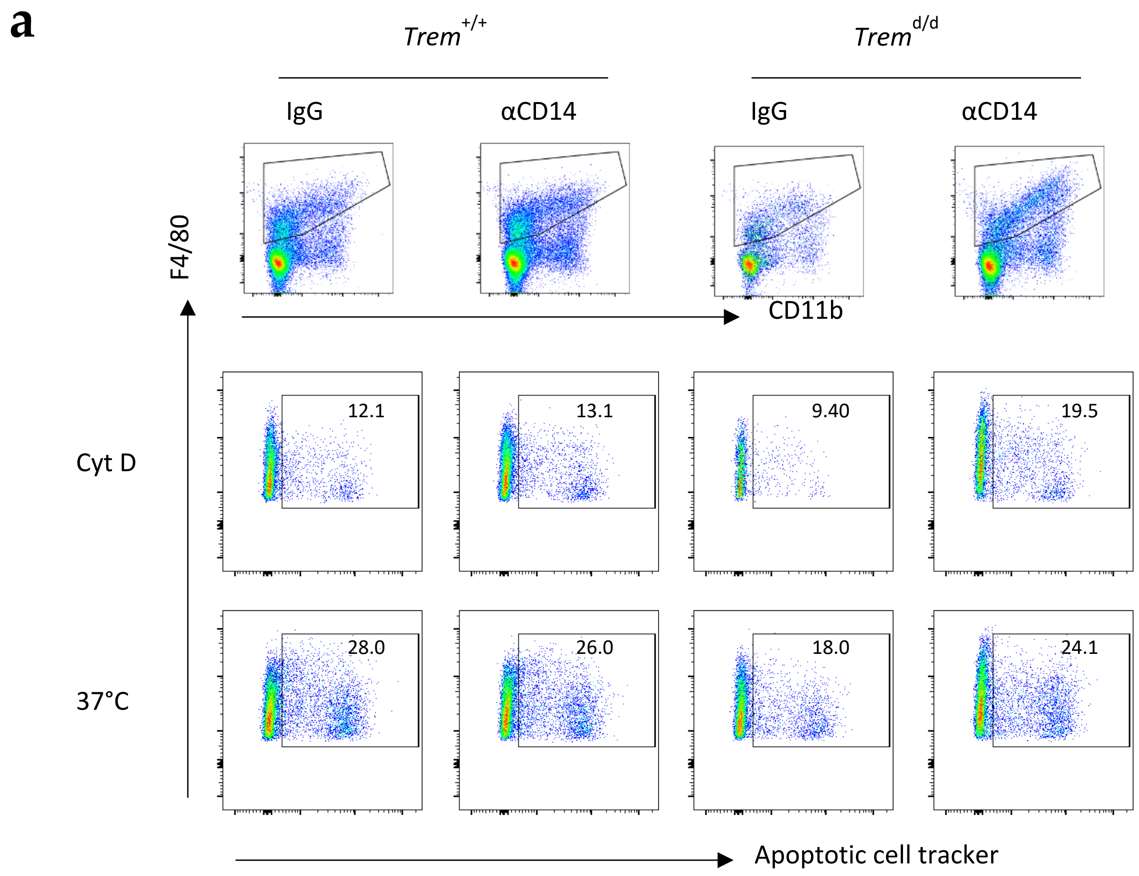

Figure S1. Synergy between TREM2 and CD14 regulates phagocytosis of apoptotic hepatocytes by liver macrophages. (**a**) Hepatic macrophages were isolated from WT (*Trem2*$^{+/+}$) and *Trem2*$^{-/-}$-mice, pre-treated with control (IgG) or anti-CD14 antibody (aCD14), stimulated with LPS for 20h, and incubated with labeled apoptotic hepatocytes. (Upper panel) Gating strategy and (bottom panel) dot plots reporting frequency of macrophages binding (Cyt D), or binding and taking up (37° C) apoptotic cells are reported.

a

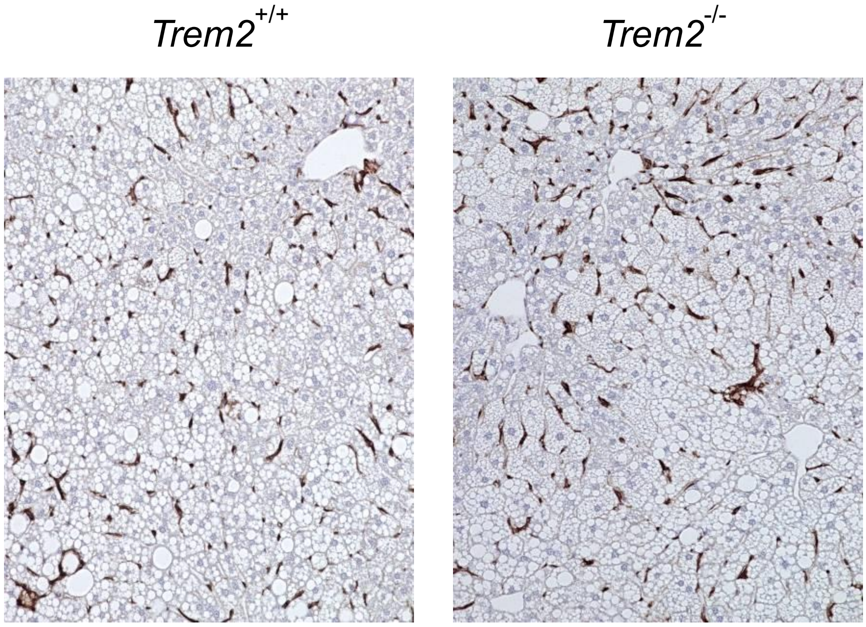

Figure S2. Hepatic macrophage infiltration after HFD feeding. (a) Representative immunoistochemical images of F4/80 antigen in liver tissue sections from WT- (Trem2+/+) and Trem2-/- - mice fed a HFD for 16 weeks, are shown.
